# BUILDING AN AGE-FRIENDLY UNIVERSITY: AN INTERGENERATIONAL CAREGIVER EDUCATION PROGRAM

**DOI:** 10.1093/geroni/igz038.3386

**Published:** 2019-11-08

**Authors:** Becky Cordes, Clare C Luz, Fei Sun, Jaewon Lee

**Affiliations:** 1 Michigan State University, East Lansing, Michigan, United States; 2 Michigan State University, East Lansing, United States

## Abstract

A rapidly aging U.S. population means an increased need for supportive services that allows aging in place. Eighty percent of such support is provided by unpaid family caregivers, many of whom are juggling multiple obligations. There is clear evidence that mental health risks of caregiving include social isolation, depression and identity loss, and potentially “caregiver burnout”. This study focuses on the lesser known impact of caregiving on college students who care for older family members and if their experiences differ from older caregivers. Presumably, students face additional stressors related to their age and school demands. Further, we posited that learning more about the aging process and interfacing with older retirees who are family caregivers through an intergenerational educational program would benefit both populations. Hosted by a campus organization, AgeAlive, which is dedicated to building an Age-Friendly University, the team’s specific aims included: 1. Conducting focus groups to identify concerns among college student and retiree family caregivers related to aging, caregiving, dementia, and intergenerational relationships. 2. Developing and piloting an intergenerational, educational program with the objective of addressing these concerns, and 3. Evaluating the program’s impact on measurable outcomes such as perceptions of aging, caregiver stress, and knowledge of resources. Focus group findings indicate that there are both shared concerns and divergent perspectives that provide opportunities for cross-generational learning. All program participants reported improved knowledge of aging, communication skills, and resources that will have a direct, positive impact. Lessons learned can guide development of supportive services for student and retiree caregivers.

